# Preventive and Therapeutic Interventions in Solar Elastosis and Photoaging: A Comprehensive Systematic Review

**DOI:** 10.3390/biomedicines13112758

**Published:** 2025-11-11

**Authors:** Francesco Leonforte, Tiziano Pergolizzi, Vito Nicosia, Fabio Nicoli, Giovanni Genovese, Cristina Genovese, Kidakorn Kiranantawat, Rosario Perrotta, Antonio Mistretta

**Affiliations:** 1 Department of Integrated Hygiene, Organizational, and Service Activities (Structural Department), Health Management, University Hospital Polyclinic “G. Rodolico—San Marco”, 95123 Catania, Italy; 2 Department of General Surgery and Medical-Surgery Specialties, University of Catania, 95123 Catania, Italy; tizianopergolizzi@gmail.com (T.P.); dr.fabionicoli@gmail.com (F.N.); r.perrotta@unict.it (R.P.); 3 Department of Medical, Surgical Sciences and Advanced Technologies “G.F. Ingrassia”, University of Catania, 95123 Catania, Italy; anmist@unict.it; 4 Translational Molecular Medicine and Surgery, University of Messina, 98122 Messina, Italy; gigenovese@unime.it; 5 Department of Biomedical, Dental and Morphological and Functional Imaging Sciences, University of Messina, 98122 Messina, Italy; crigenovese@unime.it; 6 Division of Plastic and Maxillofacial Surgery, Ramathibodi Hispital, Mahidol University, Bangkok 10400, Thailand; kidakorn.plasticsurgery@gmail.com; 7 Scientific Communication Service, National Institute of Public Health (Istituto Superiore di Sanità), 00161 Roma, Italy

**Keywords:** photoaging, skin rejuvenation, prevention, regenerative medicine, laser therapy, aesthetic surgery, preventive dermatology, photoprotection, UV damage, public health

## Abstract

**Background/Objectives:** Solar elastosis, a key histopathological alteration in skin photodamage, results from chronic UV exposure and photoaging. Clinically, it manifests as deep wrinkles, laxity, and a dull complexion. The growing demand for effective treatments has spurred the development of numerous therapeutic strategies. This systematic review aims to synthesize and critically evaluate the scientific evidence regarding interventions for treating the clinical and histological manifestations of solar elastosis, to provide an updated overview and guide future clinical practice. **Methods:** PubMed, Scopus, ProQuest, and Web of Science databases were searched for articles published in the last ten years. Clinical studies on adults with signs of solar elastosis and photoaging, evaluating therapeutic interventions, were included. Primary outcomes were clinical and histopathological improvements, while secondary outcomes included skin elasticity, safety, and patient satisfaction. This review was registered in the PROSPERO database under registration number CRD420251086680. **Results:** Twenty-two studies, totaling 608 participants, were included. The analyzed therapies comprised a wide range of strategies, including energy-based devices (laser, radiofrequency), stem cell derivatives, bioactive topical compounds, and growth factor-rich plasma. Device-assisted and biologically augmented interventions consistently improved visible photoaging outcomes and skin elasticity, with selective histologic remodeling, heterogeneous effects on barrier function, and an overall acceptable safety profile, with mild and transient adverse events. Patient satisfaction was consistently high. **Conclusions:** Therapeutic strategies in solar elastosis and photoaging, particularly those combining energy-based devices with regenerative agents, have proven effective in improving the structural and functional aspects of photodamaged skin. Although the results are promising, the current literature is limited by methodological heterogeneity and small sample sizes. High-quality randomized controlled trials with long-term follow-up are needed to establish standardized, evidence-based protocols.

## 1. Introduction

Skin aging is an inevitable biological process driven by a combination of intrinsic (genetic) and extrinsic (environmental) factors [1]. Among the latter, chronic and cumulative exposure to ultraviolet (UV) radiation from sunlight is the principal cause of an accelerated and distinct form of aging known as photoaging [2,3]. It is estimated that a substantial proportion of visible facial aging results from sun exposure and other environmental influences collectively termed the “exposome” [4]. Beyond aesthetic concerns, photoaging represents an important public health issue, as it is the leading risk factor for the development of precancerous lesions, such as actinic keratosis, and skin cancers, including basal cell and squamous cell carcinoma [5,6].

At the molecular level, solar elastosis arises from UV-driven dysregulation of the dermal elastic matrix: elastin/tropoelastin synthesis is increased, but imbalanced microfibrillar cofactors—reduced Latent TGF-beta binding protein (LTBP)-4 with increased fibrillin-1, LTBP-2, and fibulin-4—promote aberrant fiber assembly and the deposition of thick, disorganized elastotic material. At the same time, a protease–antiprotease imbalance (higher matrix metalloproteinases (MMP)-12 and neutrophil elastase activity alongside elafin-mediated protection of elastin) and extracellular-matrix disruption—versican cleavage, hyaluronan loss, and collagen alterations—undermine viscoelastic integrity. Age-related declines in elastolysis (for example, reduced cathepsin K activity) further impede clearance, consolidating extracellular accumulation [7,8].

The histopathological hallmark of advanced and chronic actinic damage is solar elastosis, characterized by the massive accumulation of abnormal, aggregated, and dysfunctional material composed of degraded tropoelastin, fibrillin, and other matrix proteins within the papillary and mid-reticular dermis [9]. This elastotic material disrupts the normal dermal architecture. Clinically, these structural alterations manifest as deep wrinkles, loss of elasticity and firmness, skin laxity, and a yellowish complexion [10].

The growing demand for effective treatments to reverse or mitigate these signs has driven the development of an expanding dermatological therapeutic arsenal. In addition to established treatments like topical retinoids [11], the current therapeutic landscape includes energy-based devices, such as ablative and non-ablative fractional lasers and microneedling radiofrequency (MFR), which induce zones of controlled thermal injury to stimulate a healing response and collagen remodeling [12,13]. In parallel, regenerative medical approaches have gained prominence, employing biological agents that leverage endogenous cellular and molecular mechanisms to enhance wound healing, reduce inflammation, and restore skin structure and function [14,15]. Despite this broad range of therapeutic strategies, evidence regarding their capacity to correct the histological alterations of photoaging remains limited and fragmented. Many studies relied on subjective clinical assessments and exhibited significant heterogeneity in designs, protocols, and outcomes measured, impeding direct comparison and the formulation of evidence-based recommendations. Moreover, previous studies have not systematically compared the correspondence between histological evidence and clinical outcomes.

Therefore, this systematic review aims to synthesize and critically evaluate the existing scientific evidence on innovative therapeutic interventions for both the clinical and histopathological manifestations of solar elastosis and photoaging. Through rigorous analysis of efficacy, safety profiles, and patient-reported outcomes, this work seeks to provide a comprehensive overview of the current state of knowledge, identify gaps in the scientific literature, and guide future research and clinical practice in the management of UV-induced skin damage.

## 2. Materials and Methods

### 2.1. Overview

This systematic review was conducted in accordance with the PRISMA guidelines [16]. The protocol for this systematic review has been officially registered in the international PROSPERO database (CRD420251086680).

### 2.2. Search Strategy and Study Selection

PubMed, Scopus, ProQuest, and Web of Science online databases were used to generate potentially relevant articles through structured search queries, developed by combining relevant keywords, synonyms, and, where applicable, medical subject headings (MeSH) terms. The use of Boolean operators and truncation techniques optimized the search strings, ensuring comprehensive retrieval of the current scientific literature. The detailed full search strategy, adapted to the syntax and search requirements of each database, is available in Appendix A. Database-specific filters were applied to refine the search. These were used to exclude non-relevant study designs and publication types, and to limit the results to studies published from January 2014 onward that were in the English language (see Supplementary Materials, Tables S1 and S2). The research was completed in April 2025.

### 2.3. Eligibility Criteria

Inclusion and exclusion criteria were defined before the screening process started according to the PICO-S framework:

- Population (P)

Studies involving human adults (≥18 years) with clinical or histopathological features of photoaging and solar elastosis.

- Intervention (I)

Damage prevention and innovative therapeutic interventions against UV-induced dermal injury.

- Comparator (C)

Studies comparing intervention(s) to prevention, placebo, or conventional treatment. Where a comparator is absent, the study design should be clearly recognizable, and the absence of a comparator should be methodologically justified.

- Outcomes (O)

Primary Outcomes: improvement in clinical or histopathological measures of photoaging (e.g., validated clinical scales, digital imaging assessments, and histological features).

Secondary Outcomes: skin elasticity is quantified by clinical or biomechanical instruments, patient-reported outcomes and quality-of-life scores, incidence of adverse events and safety endpoints, and precancerous lesions onset.

- Study Designs (S)

Observational studies (e.g., cross-sectional, cohort, or case–control), experimental trials (e.g., randomized controlled trials, non-randomized interventional studies).

The following types of studies were excluded:

- Animal or in vitro studies.
- Reviews, editorials, commentaries, letters to the editor, study protocols without original data, abstracts, case reports, or case series.
- Studies not published in English.

### 2.4. Screening

**Figure 1:**
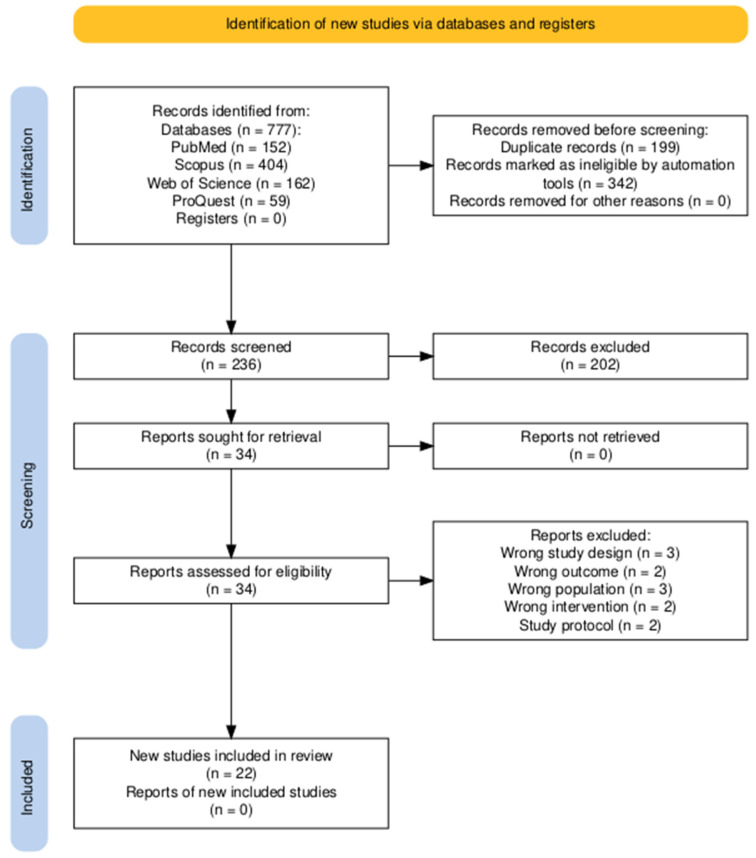
PRISMA flow diagram illustrating the study selection process. A total of 777 records were identified across the databases, with 541 removed before screening. Of the 236 records screened, 34 were assessed for eligibility, and 22 studies were ultimately included in the review.

The study selection process was conducted using the Rayyan^©^ platform. First, duplicate entries were deduplicated using an integrated tool. Subsequently, titles and abstracts were independently screened in accordance with the eligibility criteria by two reviewers in a blinded fashion (V.N. and T.P.), with any disagreements resolved through consultation with a third reviewer (F.L.). In the same way, full-text screening was conducted on all articles deemed potentially eligible in the same way. PRISMA flowchart of the screening process is shown in Figure 1 [17].

### 2.5. Data Extraction and Synthesis

A standardized Microsoft^®^ Excel 365 data extraction form was purposely developed. The extraction process was performed by two researchers (V.N. and T.P.), and disagreements were resolved through consultation with a third (A.M.). The following relevant information was extracted from each study: author and year of publication, country where the study was conducted, study design, sample size and key characteristics, intervention type and details, outcomes evaluated, limits, conclusion, and implications.

A qualitative synthesis of the data evidence presented in individual studies was conducted due to their heterogeneity. The approach integrated both narrative and tabular synthesis and was guided by thematic analysis principles, focusing on primary and secondary outcomes and allowing for the identification of recurring patterns across the studies. Studies were selected and organized according to their principal outcomes to maintain thematic coherence, notwithstanding the substantial variability among individual study designs and characteristics. Primary outcomes included clinical and histopathological changes in photoaging and solar elastosis features, measured by validated assessment scales, imaging techniques, and histological analysis of dermal elastic fiber organization. Secondary outcomes included skin elasticity, assessed with biomechanical devices, quality of life, measured via standardized questionnaires, patient-reported outcomes, addressing appearance and psychosocial impact, frequency and severity of adverse events, and incidence of precancerous lesions.

### 2.6. Risk of Bias Assessment

The methodological quality was assessed by employing appropriate tools consistently with the design of individual studies. In RCTs, risk of bias was evaluated through the RoB 2.0 (risk-of-bias tool for randomized trials) tool, developed by Cochrane to appraise five domains, including randomization, deviations from intended interventions, missing outcome data, measurement of outcomes, and selection of reported results. Each domain was rated using signaling questions, leading to an overall judgment of “low risk”, “some concerns”, or “high risk” of bias [18].

In non-randomized experimental studies, risk of bias was evaluated through the ROBINS-I (Risk of Bias in Non-randomized Studies of Interventions) tool, assessing seven domains, including confounding, selection of participants, classification of interventions, deviations from intended interventions, missing data, outcome measurement, and selection of reported results. Judgments are made across each domain to determine an overall risk of bias rating: “low”, “moderate”, “serious”, “critical”, or “no information” [19].

Quality appraisal was performed by two researchers (V.N. and F.L.), and any discrepancies were resolved by a third (A.M.). Two separate graphical representations of the risk of bias of included studies that were prepared using the robvis (2025) tool [20].

## 3. Results

### 3.1. Overview of Included Studies

A total of 22 studies published between 2015 and 2025 were included, comprising 608 participants (see Table 1) [21,22,23,24,25,26,27,28,29,30,31,32,33,34,35,36,37,38,39,40,41]. Specifically, studies were published in 2015 (*n* = 2), 2016 (*n* = 4), 2017 (*n* = 2), 2019 (*n* = 2), 2020 (*n* = 3), 2021 (*n* = 3), 2023 (*n* = 4), 2024 (*n* = 1), and 2025 (*n* = 1). The mean age of participants, calculated as the average of reported mean ages, was approximately 49.3 years. Participants were predominantly female (64.1%), whereas male participants were explicitly mentioned in only a minority of studies.

Studies originated primarily from the United States (*n* = 4) and Indonesia (*n* = 4), followed by Spain (*n* = 3), Brazil (*n* = 2), China (*n* = 3), and Poland (*n* = 2). Other countries, including Sweden, Italy, Singapore, and Egypt, were represented by a single study each.

The methodologies encompassed randomized controlled trials (*n* = 8), including four with a split-face design; non-randomized, non-controlled (pre-post) experimental studies (*n* = 8); and non-randomized controlled trials (*n* = 6), of which two also adopted a split-face design. Sample sizes varied considerably, ranging from 9 to 60 participants per study, with evaluation periods extending from 3 days to 36 months post-treatment.

The included studies covered the whole range of Fitzpatrick skin types (I–VI). The overall distribution was skewed toward skin types I–IV, reflecting predominant Caucasian and East Asian populations (*n* = 5). Only one study included participants across the full spectrum (types I–VI), focusing particularly on types I-III.

A smaller number of studies reported types III–IV (*n* = 3) and II–V (*n* = 3). Only one study explicitly encompassed type VI, indicating limited representation of darker skin tones. The remaining ten studies did not specify Fitzpatrick type.

Interventions included a broad spectrum of regenerative strategies, such as biologically active topicals (e.g., snail mucin derivatives, conjugated linolenic acid), mesotherapy-assisted delivery of bioactive compounds, stem cell derivatives, laser-assisted drug delivery, and energy-based devices (e.g., fractional CO_2_ laser, sublative fractional radiofrequency). Further details about interventions are provided in Table 2. Histological confirmation via skin biopsies was performed in seven studies. All studies assessed multidimensional endpoints, including skin structure, elasticity, patient-reported outcomes, and adverse event profiles.

**Figure 2:**
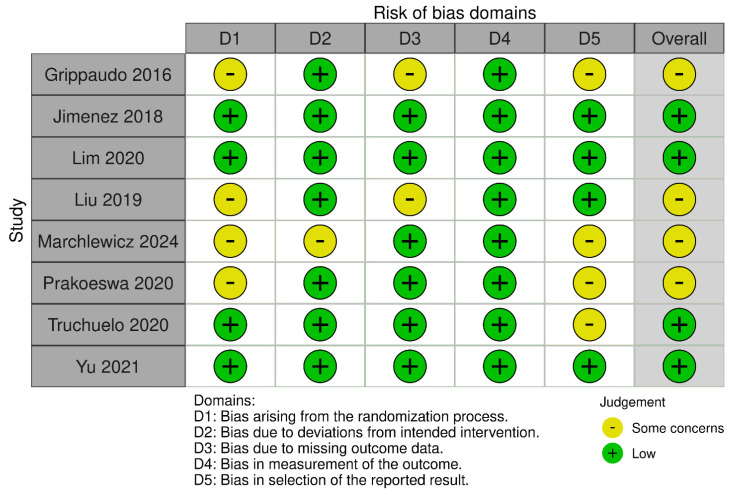
Risk of bias for randomized and split-face controlled trials (RoB 2). Judgements across five domains (D1–D5) and the overall rating are shown for each study (green, low; yellow, some concerns). Most trials showed low risk in D2, D4, and D5, with recurrent “some concerns” for the randomization process (D1), missing outcome data (D3), and selection of the reported results (D5) [26,28,29,30,32,34,37,42].

**Figure 3:**
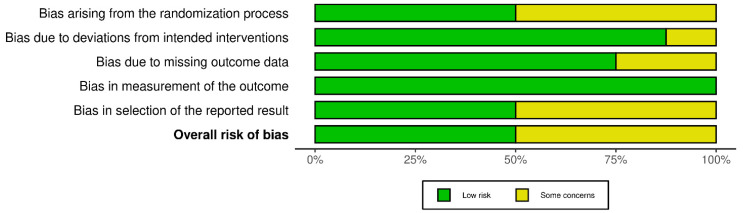
Summary of risk-of-bias assessments for randomized and split-face controlled trials (RoB 2). Stacked bars display the proportion of studies rated low risk (green) or some concerns (yellow) for each domain (D1–D5) and for the overall judgment. Concerns most frequently involved the randomization process (D1) and missing outcome data (D3), while other domains were predominantly low risk.

**Figure 4:**
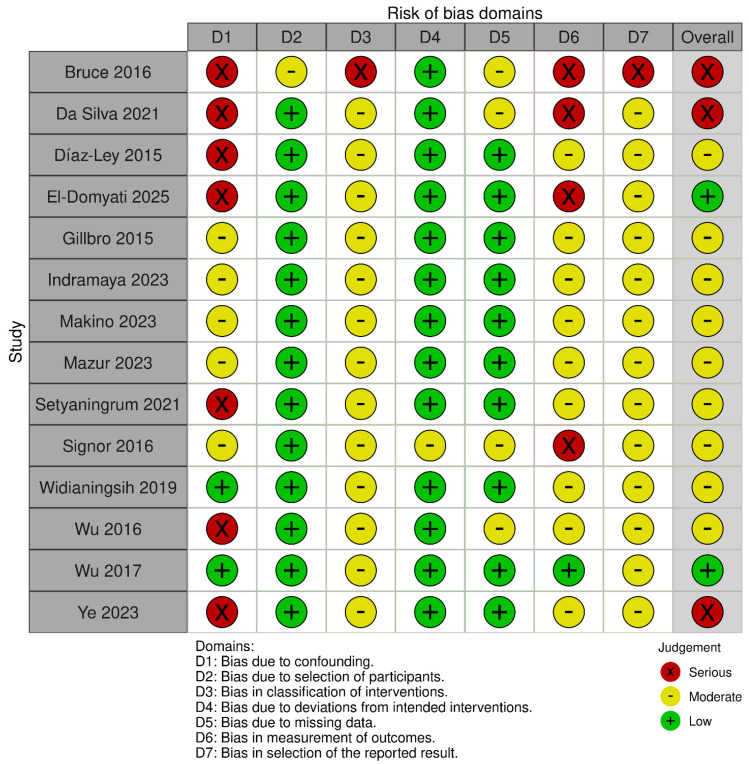
Risk of bias for non-randomized studies of interventions (ROBINS-I). Judgements across seven domains (D1–D7) and the overall rating are shown for each study (green, low; yellow, moderate; red, serious). Risk due to confounding (D1) and classification of interventions (D3) was frequently moderate–serious, whereas deviations from intended interventions (D4) and measurement of outcomes (D6) were generally low; selection-related domains (D2, D7) and missing data (D5) varied across studies [21,22,23,24,25,27,31,33,35,36,38,39,40,41].

**Figure 5:**
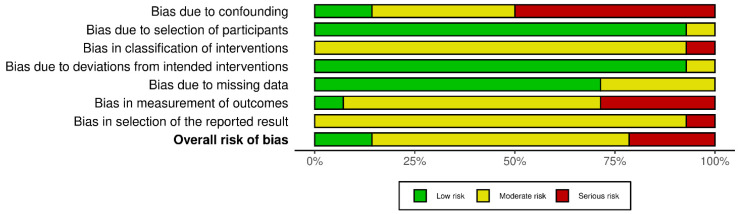
Summary of risk-of-bias assessments for non-randomized interventional studies (ROBINS-I). Stacked bars display the proportion of studies rated low (green), moderate (yellow), or serious (red) risk for each domain (D1–D7) and for the overall judgment. Elevated risk was most evident for confounding (D1), classification of interventions (D3), and missing data (D5); deviations from intended interventions (D4) and outcome measurement (D6) were generally low to moderate.

Regarding risk of bias, eight randomized trials and fourteen non-randomized studies were appraised using RoB 2 and ROBINS-I tools, respectively (see Figure 2, Figure 3, Figure 4 and Figure 5). In the randomized trials, the predominant risks of bias arose from inadequately reported randomization procedures and allocation concealment, together with the absence of preregistered protocols or prespecified analyses, which raised concerns about selective reporting (e.g., Grippaudo et al. [26]; Liu et al. [30]; Prakoeswa et al. [34]; Jiménez et al. [28]; Lim et al. [29]; Truchuelo et al. [37]; Yu et al. [42]; and Marchlewicz et al. [32]). Missing outcome data was uncommon but not negligible (e.g., four withdrawals in Liu et al. [30]; two discontinuations in Grippaudo et al. [26]), and deviations from intended interventions were infrequent. These features collectively support “some concerns” ratings for several domains despite otherwise sound trial conduct.

Across the non-randomized studies, most were judged to have an overall moderate risk of bias, whereas several were rated as having a serious (and, in one instance, critical) overall risk. The principal drivers were uncontrolled baseline confounding—often in single-arm designs—misclassification of interventions, non-blinded and subjective outcome assessment, and the absence of preregistration/prespecified analyses. These limitations were characteristic of studies such as Díaz-Ley et al. [23], Wu et al., 2016 [40], Ye et al. [41], Setyanigrum et al. [35], Da Silva et al. [22], and El Domyaty et al. [24]; selection from a prior cohort contributed to a critical overall rating for Bruce et al. Where intra-subject controls were used or procedures were more clearly specified (e.g., Gillbro et al. [25], Indramaya et al. [27], Makino et al. [31], Mazur et al. [33], Widianingsih et al., 2019 [38]), overall risk tended to be moderate. The lowest risk among the non-randomized body was observed in a non-randomized, blinded trial (Wu et al., 2017 [39]), although the lack of preregistration still limited its appraisal.

### 3.2. Clinical and Histopathological Improvement in Photoaging and Solar Elastosis

Across six included studies spanning photodynamic therapy (PDT), microneedling-based approaches [with needle-free mesotherapy, sublative fractional radiofrequency (SFRF), or radiofrequency (RF) alone], autologous plasma-rich in growth factors (PRGF), and topical 1% acetyl aspartic acid, structural and histopathologic improvements in photoaging were evident [23,24,25,30,32,33]. The most consistently replicated outcomes were reduced solar elastosis (3/6 studies: PDT, microneedling + SFRF, PRGF) [23,24,33] and enhanced collagen quality/quantity across the epidermal–dermal unit (4/6 studies: thicker dermal bundles, normalized collagen I, improved organization, and increased collagen IV/fibrillin-1) [24,25,32,33]. Specifically, Mazur et al. (2023) [33] reported that, in three months post-PDT, the prevalence of solar elastosis decreased from 95.6% to 92% in classic actinic keratosis (AK) and from 60% to 52% in pigmented AK, with concomitant reductions in disorganized spinous layers and inflammatory infiltrates on reflectance confocal microscopy. Dermoscopically, PDT led to complete resolution of the “rhomboidal pattern” in both classic and pigmented AK; the “starburst pattern” and “jelly sign” in classic AK; and the inner gray halo, “rosette sign,” and central crust in pigmented AK. Marchlewicz et al. (2024) [32] observed reduced epidermal thickness, a 30% increase in dermal collagen bundle thickness, enhanced fibroblast activation, and an upregulation of dermal remodeling markers (Ki-67, PCNA, MMP-2) three months after treatment with microneedling and needle-free mesotherapy using *Helix aspersa* snail mucus filtrate. El-Domyati et al. (2025) [24] reported normalized collagen I expression (*p* = 0.001) and reduced elastotic material (*p* = 0.0005) following three consecutive sessions of combined microneedling and SFRF at 1-month intervals. Significant improvements were also noted in skin tightening, in texture, and in rhytides. Díaz-Ley et al. (2015) [23] and Liu et al. (2019) [30] found significant histopathological improvements following treatment with PRGF and microneedling RF (MRF), respectively. PRGF increased epidermal and papillary dermal thickness (*p* < 0.001), fibroblast density (*p* < 0.001), and collagen organization (*p* < 0.05), and reduced the average area fraction of solar elastosis (*p* < 0.05). MFR produced improvements according to the Global Aesthetic Improvement Scale at the 1- and 3-month follow-up visits. Gillbro et al. (2015) [25] reported significant increases in collagen IV (+13.2%) and fibrillin-1 (+6.4%) after treatment with 1% acetyl aspartic acid (AAA) compared with the vehicle.

Prakoeswa et al. (2020) [34], Setyaningrum et al. (2021) [35], and Indramaya et al. [27] (2023) investigated amniotic membrane stem cell derivatives, combined with topical vitamin C. Notably, Prakoeswa et al. (2020) [34] demonstrated significant improvements in wrinkles (*p* = 0.008), UV spots (*p* = 0.046), and pore appearance (*p* = 0.046) after treatment with topical amniotic membrane stem cell metabolite product (AMSC-MP) plus vitamin C delivered via Dermapen^®^ microneedling, compared with AMSC-MP alone. Lim et al. (2020) [29] reported significant improvements in transepidermal water loss (TEWL), skin roughness, and firmness using a combined regimen of *Cryptomphalus aspersa* secretion filtrate with fibroblast growth factor-like activity (SCA^®^) and *Cryptomphalus aspersa* egg extract with skin stem cell activity (IFC-CAF^®^) regimen versus placebo. Makino et al. (2023) [31] reported clinical improvements following combined diamond-tip microdermabrasion and hyaluronic acid serum (HA^5^-DG). Early gains included reductions in fine lines/wrinkles and improvements in dryness, smoothness, radiance, firmness, and hydration, whereas later benefits encompassed decreases in coarse wrinkles, better tone, reduced hyperpigmentation and photodamage, and lower TEWL.

Six studies considered clinical effects related to laser-based approaches [27,28,37,38,39,43]. Across fractional CO_2_, erbium:YAG, and picosecond alexandrite platforms, laser-assisted delivery of bioactive topicals (e.g., amniotic membrane derivatives, regenerative serums, and SCA-based cosmeceuticals) generally yielded superior or additive clinical benefits versus comparator methods. Reported gains clustered around dyschromia, texture, pores, and rhytides, with supportive histologic signals and variable durability across endpoints. Indramaya et al. [27] reported superior improvements in wrinkles, UV spots, and pore appearance with fractional CO_2_ laser-assisted delivery of amniotic membrane stem cell-conditioned medium (AMSC-CM) plus vitamin C, compared with microneedling-assisted delivery. Widianingsih et al. (2019) [38] likewise demonstrated comparable improvements using fractional erbium:YAG laser-assisted transdermal delivery of topical AMSC-MP. Wu et al. (2017) [39] observed clinical elastosis reduction following topical conjugated linolenic acid after fractionated CO_2_ laser resurfacing. Wu et al. (2016) [40] documented significant improvements (*p* < 0.05) at 1- and 3-month follow-up in decolletage photodamage (dispigmentation, keratosis, and skin texture), after 755 nm picosecond pulsed alexandrite laser (PSAL) with a diffractive lens array (DLA). Improvements in rhytides were transient and not sustained at 3 months (*p* = 0.08), and erythema showed no meaningful change. Likewise, Yu et al. (2021) [42] described improvements in dyschromia, texture, and rhytides up to 36 months after PSAL with DLA. Jiménez et al. (2018) [28] documented increases in epidermal thickness (+20%), dermal thickness (+41%), cellular proliferation rate (+41%), and elastin density (+116%), together with improvement in static wrinkles (−42%, *p* < 0.01) 60 days after regenerative serum post YAG laser therapy. Truchuelo et al. (2020) [37] demonstrated clinically and dermoscopically a reduction in the density of microcolumns in the treated hemiface compared to the vehicle-treated side after non-ablative laser with *Cryptomphalus aspersa* secretion (SCA)-based cosmeceuticals treatment.

Two studies addressed therapeutic strategies based on chemical compounds [21,41]. Ye et al. (2023) [41] showed reduced wrinkles and improved texture after twice-daily supramolecular 0.1% retinol application in Chinese participants, albeit without histological confirmation. Bruce et al. (2016) [21] reported improvements in fine lines, coarse wrinkles, skin roughness, brown spots, and skin discoloration, along with the Physician’s Global Assessment (54% mild, 8% moderate, and 38% marked) at week 12 following initial chemical resurfacing with AGE (trichloroacetic acid, pyruvic acid, salicylic acid, mandelic acid, and lactobionic acid) and MELA (mandelic acid, potassium azeloyl diglycinate, retinol, salicylic acid, phytic acid, lactobionic acid, and lactic acid) protocols and subsequent daily skincare treatment with Phytochromatic MD^®^ Complex (lipoosomal complex of sodium copper chlorophyllin), compared with baseline evaluation.

In three studies, no statistical significance was found [22,26,36]. Signor et al. (2016) [36] identified non-significant but consistent trends toward increased collagen deposition after stromal vascular fraction (SVF) injections compared with conventional calcium hydroxyapatite filler. Da Silva et al. [22] found no significant improvement after lyophilized platelet-rich plasma (PRP) therapy compared with saline solutions. Grippaudo et al. (2016) [26] found no significant differences in skin healing time between groups treated with ionic hydrogel, either alone or in combination with clarithromycin, following fractional CO_2_ laser treatment.

Ultimately, across the included studies, the most consistent improvements emerged for clinically visible photoaging outcomes—namely, pore appearance, dyschromia/spots, rhytides, skin texture, and composite photoaging scores. Biophysical readouts broadly mirrored these trends, with several studies documenting gains in skin elasticity. Structural correlates were observed in six studies, whereas changes in cellular proliferation indices, dermoscopic features, and markers of solar elastosis were less consistent. A graphical representation of the associations found between the different therapeutic approaches and the outcomes considered is shown in Table 3.

### 3.3. Skin Elasticity and Functionality

Eight studies documented improvements in skin elasticity, most notably with energy-based modalities [22,25,27,29,30,35,37,41]. Elasticity was quantified by bio-instrumental measurements (e.g., Cutometer^®^) in five studies and by clinical assessments in the remaining studies.

Truchuelo et al. (2020) [37] reported an 11% improvement in Cutometer-derived elasticity (*p* = 0.037) following SCA-based treatments. Ye et al. (2023) [41] reported increases in R2 and R7 (+10.8%, +10.9%), reflecting augmented elasticity and a reduction in F4 (−6.1%), consistent with improved firmness, after supramolecular retinol. Gillbro et al. (2015) [25] similarly documented a 15.8% decrease in F4 after 28 days of AAA treatment (*p* < 0.05), which is comparable to retinol (14.7%) and significantly different from vehicle. Lim et al. (2020) [29] also found significant gains in elasticity (*p* < 0.05) with SCA^®^ plus IFC^®^-CAF treatment. Liu et al. (2019) [30] reported complementary improvements in skin roughness (Sa values, *p* < 0.05) after MFR versus the untreated side.

Fractional CO_2_ laser-based interventions yielded consistent improvements in elasticity, albeit without standardized mechanical assessments [27,35]. Da Silva et al. (2021) [22] observed no changes in biomechanical parameters with PRP.

### 3.4. Patient-Reported Outcomes

Across eight studies, patient satisfaction consistently favored active treatments [24,28,29,31,37,39,42,43]. El-Domyati et al. (2025) [24] recorded high satisfaction (86–97%; *p* < 0.001) after combined microneedling and SFRF therapy. Lim et al. (2020) [29] and Truchuelo et al. (2020) [37] reported superior Patient Global Assessment (PGA) scores (*p* < 0.05), associated with SCA^®^ + IFC^®^-CAF and non-ablative fractional laser + SCA^®^, respectively. Yu et al. (2021) [42] recorded 70% satisfaction at 36 months post-PSAL with DLA, while Makino et al. (2023) [31] noted up to 100% perceived improvement and 95.7% satisfaction at 12 weeks after diamond-tip microdermabrasion plus HA^5^-DG serum. Wu et al. (2017) [39] reported significant reductions in patient-reported itching and faster perceived recovery (*p* < 0.05) after topical conjugated linolenic acid following fractionated CO_2_ laser resurfacing. Jiménez et al. (2018) [28] demonstrated greater subject-perceived improvement on the regenerative-serum-treated side than on the placebo-treated side.

Three studies showed heterogeneous results. Díaz-Ley et al. (2015) [23] found that, of nine patients treated with PRGF, two were very satisfied, five were satisfied, and two were indifferent. In the study by Bruce et al. (2016) [21], 8% of participants reported no improvement after AGE/MELA treatment, 31% reported mild improvement, 31% reported moderate improvement, 23% marked improvement, and 7% provided no assessment. Wu et al. (2016) [40] reported moderate satisfaction with PSAL with DLA, with a mean rating of 2.8/6 (1 = extremely satisfied; 6 = extremely dissatisfied).

### 3.5. Safety and Adverse Events

Adverse events were predominantly mild, transient, and self-limited, with no long-term sequelae. Common side effects of laser- and energy-based treatments included erythema, edema, itching, mild pain, discomfort, and local irritation [24,26,28,30,37,38,39,40,42]. Truchuelo et al. (2020) [37] reported significantly greater reductions in erythema, tightness, and burning sensations after non-ablative fractional laser on the SCA-treated side compared with the vehicle-treated side. Yu et al. (2021) [42] observed erythema and edema following PSAL with DLA, along with post-inflammatory hyperpigmentation in a patient with baseline melasma. Without local anesthesia, the mean patient-reported pain score was 5.3 ± 0.6 out of 10.

Lim et al. (2020) [29] noted reduced stinging and erythema with snail extract (*p* < 0.05) compared to vehicle, without differences in itching, desquamation, or edema. Isolated cases of contact dermatitis and herpes zoster, unrelated to treatment, were recorded.

Makino et al. (2023) [31] reported transient facial erythema between weeks 6 and 12 post-HA^5^-DG and a case of back muscle spasm likely unrelated to treatment.

Wu et al. (2016) [40] noted one urticarial reaction.

Regarding stem cell-based treatments, Signor et al. (2016) [36] observed transient local hematoma in the SVF group.

In general, tolerability was highest with snail mucin, SCA^®^, and topical regenerative approaches [21,25,29,32,34,39]. Six studies did not report significant adverse events [21,23,25,27,32,41].

### 3.6. Precancerous Lesions Onset

Only Mazur et al. (2023) [33] evaluated precancerous lesions (actinic keratosis), demonstrating significant regression in dermoscopic and RCM features following PDT, with resolution of high-risk dermoscopic markers. No study reported increased incidence of new precancerous lesions or malignant transformation during follow-up.

**Table 2:** Overview of intervention categories and representative modalities assessed in the included studies, with corresponding references. Interventions are grouped as energy-based devices, radiofrequency and microneedling, plasma- and cell-based injectables, stem cell-derived injectables, topical cosmeceuticals and retinoids, chemical resurfacing, and mechanical resurfacing (Abbreviations: AGE, trichloroacetic acid, pyruvic acid, salicylic acid, mandelic acid and lactobionic acid; AK, actinic keratosis; AK, actinic keratosis; AMSC-CM, amniotic-derived mesenchymal stem cell conditioned medium; AMSC-MP, amniotic-derived mesenchymal stem cell metabolite product; DLA, diffractive lens array; ECM; extracellular matrix; Er:YAG, erbium-doped yttrium aluminum garnet (laser); FGF, Fibroblast growth factors; HA, hyaluronic acid; IFC-CAF, *Cryptomphalus aspersa* snail egg extract with skin stem cell activity; MELA, mandelic acid, potassium azeloyl diglycinate, retinol, salicylic acid, phytic acid, lactobionic acid, and lactic acid; RF, radiofrequency; SCA, *Cryptomphalus aspersa* secretion; SVF, stromal vascular fraction; and YAG, yttrium aluminum garnet (laser)).

Intervention Type	Specific Interventions	Reference	Description
**Energy-based devices (lasers, light, ecc…)**	Photodynamic therapy	[33]	Topical photosensitizers activated by specific light to generate reactive oxygen species, targeting dysplastic keratinocytes in AK and remodeling photodamaged tissue.
	Fractional CO_2_ laser + topical conjugated linolenic acid	[39]	Ablative fractional resurfacing followed by omega-conjugated linolenic acid to modulate inflammation and dermal repair after laser injury.
	Fractional CO_2_ laser + ionic hydrogel ± clarithromycin	[26]	Post-laser wound care using an ionic hydrogel (with or without topical antibiotic) to support re-epithelialization and barrier recovery.
	755 nm picosecond alexandrite laser with diffractive lens array	[40,42]	Picosecond photothermolysis using a DLA to create laser-induced optical breakdown microzones for pigment correction and dermal remodeling.
	Fractional non-ablative laser + SCA^®^-based cosmeceuticals	[37]	Non-ablative fractional photothermolysis combined with a snail-secretion—derived regimen to enhance post-laser regeneration.
	Fractional Er:YAG laser-assisted delivery of AMSC-MP	[38]	Ablative fractional Er:YAG microchannels used to enhance transdermal penetration of amniotic membrane stem cell metabolite product.
	Fractional CO_2_ laser-assisted delivery of AMSC-CM + vitamin C	[27]	CO_2_ laser microchannels facilitating delivery of stem cell conditioned medium with ascorbic acid to photodamaged skin.
	YAG laser + regenerative serum	[28]	Laser treatment followed by topical application of a bioactive, growth-factor–rich serum to stimulate extracellular matrix renewal.
**Radiofrequency and microneedling**	Microneedling + needle-free mesotherapy with *Helix aspersa* mucus filtrate	[32]	Percutaneous collagen induction combined with transdermal delivery (needle-free) of snail-mucin filtrate rich in glycosaminoglycans and peptides.
	Microneedling + sublative fractional radiofrequency	[24]	Mechanical microinjury paired with dermal RF heating via fractionated electrodes to induce neocollagenesis and elastin remodeling.
	Microneedling radiofrequency	[30]	RF energy delivered through insulated microneedles to create controlled thermal zones in the dermis for texture and laxity improvement.
**Plasma- and cell-based injectables**	Plasma-rich in growth factors	[23]	Autologous plasma fraction enriched in platelet-derived growth factors injected to promote dermal remodeling.
	Lyophilized platelet-rich plasma	[22]	Dehydrated, reconstituted platelet concentrate intended to deliver standardized growth factors for skin rejuvenation.
	Stromal vascular fraction injections	[36]	Autologous adipose-derived SVF (mesenchymal/stromal and perivascular cells) injected to enhance regenerative pathways and matrix deposition.
**Stem cell-derived topicals**	AMSC-MP + vitamin C via microneedling (Dermapen^®^)	[34]	Topical amniotic membrane stem cell metabolite product with ascorbic acid driven through microchannels created by microneedling.
	AMSC-CM + vitamin C (topical)	[35]	Topical conditioned medium from amniotic membrane stem cells combined with antioxidant therapy for photodamage.
**Topical cosmeceuticals and retinoids**	SCA^®^ + IFC-CAF^®^ regimen (*Cryptomphalus aspersa* derivatives)	[29]	Combination of snail-secretion filtrate with FGF-like activity and egg extracts aimed at enhancing epidermal function and firmness.
	Supramolecular 0.1% retinol	[41]	Stabilized, complexed retinol designed for improved cutaneous delivery and tolerability to stimulate epidermal turnover and dermal matrix
	1% acetyl aspartic acid	[25]	Topical ECM-modulating amino-acid derivative intended to upregulate basement-membrane and elastic-fiber components
**Chemical resurfacing**	AGE/MELA chemical resurfacing + daily Phytochromatic MD^®^ Complex	[21]	Sequential medium-depth peels followed by a liposomal sodium-copper chlorophyllin complex as maintenance skincare
**Mechanical resurfacing**	Diamond-tip microdermabrasion + hyaluronic-acid serum	[31]	Controlled epidermal exfoliation coupled with immediate application of a hyaluronan-rich serum to rehydrate and smooth the skin

**Table 3:** Heatmap summarizing reported associations across studies. Cell colors indicate evidence strength: **green** denotes a statistically significant association; **yellow** indicates conflicting findings across different studies; **red** denotes a non-statistically significant association; **gray** indicates that the specific association was not evaluated in the corresponding study (Abbreviations: AMSC-CM, amniotic-derived mesenchymal stem cell conditioned medium; AMSC-MP, amniotic-derived mesenchymal stem cell metabolite product; Er:YAG, erbium-doped yttrium aluminum garnet (laser); HA, hyaluronic acid; IFC-CAF, *Cryptomphalus aspersa* snail egg extract with skin stem cell activity; IGA, Investigator Global Assessment; PGA, Physician Global Assessment; SCA, *Cryptomphalus aspersa* secretion; and YAG, yttrium aluminum garnet (laser)).

	Pore Appearance	Spots and Dyschromia	Clinical Photoaging (PGA, IGA)	Trans-Epithelial Water Loss	Healing Time	Erythema	Rhytides	Skin Texture	Epidermal Thickness	Dermal Thickness	Collagen Fiber Deposition and Organization	Cellular Proliferation rate	Histopathological Signs of Solar Elastosis	Dermoscopic Pattern	Skin Elasticity
**1% acetyl aspartic acid**															
**755 nm picosecond alexandrite laser with diffractive lens array**															
**AGE/MELA chemical resurfacing + daily Phytochromatic MD^®^ Complex**															
**AMSC-CM + vitamin C (topical)**															
**AMSC-MP + vitamin C via microneedling (Dermapen^®^)**															
**Diamond-tip microdermabrasion + hyaluronic-acid serum**															
**Fractional CO_2_ laser + ionic hydrogel + clarithromycin**															
**Fractional CO_2_ laser + topical conjugated linolenic acid**															
**Fractional CO_2_ laser-assisted delivery of AMSC-CM + vitamin C**															
**Fractional Er:YAG laser-assisted delivery of AMSC-MP**															
**Fractional non-ablative laser + SCA^®^-based cosmeceuticals**															
**Intradermal PRGF**															
**Microneedling + needle-free mesotherapy with *Helix aspersa* mucus filtrate**															
**Microneedling + sublative fractional radiofrequency**															
**Photodynamic therapy**															
**Plasma-rich in growth factors**															
**SCA^®^ + IFC-CAF^®^ regimen (*Cryptomphalus aspersa* derivatives)**															
**Stromal vascular fraction injections**															
**Supramolecular 0.1% retinol**															
**YAG laser + regenerative serum**															

## 4. Discussion

This systematic review synthesizes the evidence from the last decade on therapeutic approaches for solar elastosis and photoaging. The analysis of 22 studies reveals that a wide range of interventions—from energy-based devices to stem cell derivatives and bioactive topical compounds—can produce significant improvements in both histological and clinical outcomes. The results consistently demonstrate not only a reversal of structural damage at the dermal level but also an improvement in skin functionality and satisfaction, with generally favorable safety profiles.

### 4.1. Comparison of Intervention Mechanisms

The solid evidence of histopathological improvement in dermal damage is of crucial importance. Indeed, the primary goal of anti-aging therapies is to stimulate the endogenous repair processes of the extracellular matrix (ECM), counteracting collagen fragmentation and abnormal elastotic material accumulation through neocollagenesis and the reorganization of elastic fibers [44,45,46]:

- Energy-Based and Physical Approaches: Treatments such as radiofrequency microneedling have shown a normalization of type I collagen expression and a reduction in elastotic material, confirming the ability of these technologies to induce deep dermal remodeling [47]. Fractional lasers (both ablative and non-ablative) remain a cornerstone in the treatment of photoaging, with their effectiveness in promoting deep collagen remodeling being well-documented and serving as a benchmark [48]. Picosecond lasers offer an alternative with a potentially superior safety profile, inducing minimal thermal damage and leveraging photomechanical effects to stimulate skin regeneration [49]. The growing use of these technologies to enhance the delivery of topical drugs, a technique called laser-assisted drug delivery (LADD), represents a further frontier for maximizing therapeutic efficacy [50].
- Biological Therapies and Bioactive Compounds: The use of bioactive compounds, such as mesenchymal stem cell (MSC) derivatives or their secretomes, demonstrates a significant capacity to modulate fibroblast activity and reduce local inflammation [51]. An emblematic example is the role of hyaluronic acid, a key component of the ECM whose biological functions critically depend on its molecular weight. It has been shown that low molecular weight HA (LMWHA) fragments actively promote tissue regeneration and restore epithelial architecture in atrophic contexts, highlighting their potential as bioactive agents in dermatology [52]. This ability to modulate local inflammation is particularly relevant in the context of “inflammaging,” the chronic low-grade inflammation that characterizes the aging process and significantly contributes to tissue degradation, a phenomenon driven in part by the senescence-associated secretory phenotype (SASP) [53].

It is interesting to note that not all autologous therapies have produced equivalent results. The study by Da Silva et al. on the use of lyophilized platelet-rich plasma (PRP) found no significant improvements, in contrast to the positive evidence for PRGF [22]. This highlights a critical debate in the literature: the efficacy of PRP is highly dependent on the platelet concentration, method of activation, and injection protocol, leading to significant heterogeneity in clinical outcomes [14,54].

### 4.2. Ethnic Differences in Outcomes

Across the studies, Asian (China, South Korea, and Indonesia) and predominantly white (Europe and the USA) cohorts demonstrated broadly comparable efficacy of energy-based and adjuvant approaches, with consistent improvements in wrinkles, texture, pores, and dyschromia [55]. Nevertheless, the emphasis and risk profile differed: Asian reports more often foregrounded pigmentary outcomes (UV spots, dyschromia) with fractional and picosecond platforms, whereas several European/North American studies coupled clinical gains with histologic evidence of matrix remodeling [56]. Safety was acceptable in both groups, but the risk of post-inflammatory hyperpigmentation remains a key concern in melanin-rich phototypes, reinforcing the need for pigment-conservative parameters, staged treatments, and rigorous photoprotection [57]. Topical responses may also diverge, as Asians can exhibit greater retinoid sensitivity despite the good tolerability reported for supramolecular retinol in one Chinese cohort, supporting gradual uptitration with barrier support [58]. Overall, current evidence is underpowered for definitive between-group comparisons because there were few trials stratified by ethnicity/phototype, phototypes V–VI were sparsely represented, and several outcome tools were validated primarily on Caucasian skin.

### 4.3. Feasibility in Clinical Practice and Outcomes

The restoration of dermal structure has translated into objective improvements in the biomechanical functionality of the skin, as confirmed by studies reporting a quantifiable increase in elasticity, primarily measured with the Cutometer^®^. This instrument is widely validated for the non-invasive characterization of the skin’s viscoelastic properties, making it a gold standard for the objective assessment of treatment efficacy [59]. The observed improvements in elasticity and firmness parameters (e.g., R2, R7, F4) correlate well with histological observations of neocollagenesis and reorganization of elastic fibers, closing the loop between microscopic repair and macroscopic functional recovery. This link is fundamental, as the loss of elasticity is one of the main clinical manifestations of photoaging [60].

A crucial aspect for clinical adoption is the safety profile and patient acceptance. The reported adverse events were in line with what is expected for minimally invasive procedures: mild, transient, and self-limiting [61,62,63]. The excellent tolerability profile of regenerative topical compounds, such as snail extract or growth factor-based serums, not only makes them suitable as primary treatments but also as adjuvants to reduce post-procedural erythema and edema, thereby accelerating recovery [43]. Patient satisfaction, which is consistently high across almost all studies, underscores the importance of integrating patient-reported outcomes into clinical evaluation, since they are essential for a holistic assessment of efficacy, which also considers the impact on quality of life and self-perception, aspects that objective measurements alone cannot grasp [64,65]. A secondary outcome of great clinical relevance was the monitoring of precancerous lesions. Although only one study focused on this aspect, the demonstrated regression of actinic keratosis after PDT is consistent with the extensive literature supporting the use of this therapy for the treatment of field cancerization [66,67]. It is reassuring that none of the regenerative therapies showed an increased oncological risk, which is a fundamental safety consideration when using treatments that stimulate cell proliferation.

### 4.4. Future Research Directions and RCT Design

Although the evidence gathered is promising, the current literature is characterized by a degree of heterogeneity in protocols and evaluation methodologies. A significant limitation is the lack of robust data across diverse skin phototypes, as only one study included the full Fitzpatrick scale (types I–VI). To consolidate the current findings and establish evidence-based clinical guidelines, future research should focus on designing high-quality randomized controlled trials (RCTs) that explicitly validate the safety and efficacy of these interventions in relevant, underrepresented skin populations. Such trials should include the following:

1. Head-to-head comparisons: Directly comparing the efficacy of different therapeutic modalities (e.g., picosecond vs. fractional ablative lasers) or different formulations of biological therapies (e.g., standardized PRP preparations) to identify superior approaches.
2. Standardized outcome measures: Employing validated and objective assessment tools, such as the Cutometer^®^, alongside standardized histological and molecular analyses (e.g., quantification of type I collagen or SASP markers) to allow for reliable cross-study comparisons.
3. Long-term follow-up: Assessing the durability of clinical and histological improvements to determine the stability of results over time and the potential need for maintenance treatments.

The implementation of such rigorously designed RCTs will be fundamental to translating the innovations discussed in this review into established and effective clinical protocols for the treatment of cutaneous photoaging.

### 4.5. Limitations

To the best of our knowledge, this is the first systematic review addressing therapeutic approaches to clinical and histopathological photoaging-related phenomena. Our analysis highlights several limitations in the current literature. The methodological quality of the included studies is variable; while randomized controlled trials (RCTs) presented a predominantly low or “some concerns” risk of bias, non-randomized studies showed a higher risk of bias, especially regarding confounding and participant selection, which weakens the strength of the conclusions. The small sample sizes of many studies limit the generalizability of the results. Furthermore, the wide heterogeneity of interventions, protocols, devices, and evaluation parameters made a quantitative meta-analysis and direct comparison between therapies difficult. The duration of follow-up was also a limitation since many were limited to short periods, not allowing for the assessment of the actual duration of the benefits obtained. There is a poor representation of certain phototypes, as most studies focused on Fitzpatrick phototypes I-IV, with very limited representation of types V and VI, which constitutes a significant gap, as the response to energy-based treatments can vary greatly among them. Lastly, since this is a systematic review, it is possible that some relevant studies may have been overlooked.

## 5. Conclusions

In summary, this systematic review highlights the remarkable progress made in the last decade in the treatment of solar elastosis, underscoring that innovative strategies combining energy-based devices with potent regenerative agents show great promise.

However, the current literature is limited by methodological heterogeneity and small sample sizes, which prevent the formulation of definitive evidence-based guidelines. To translate these promising findings into established clinical protocols, future research must address these shortcomings through specific methodological improvements. It is imperative to conduct high-quality randomized controlled trials (RCTs) that incorporate the following:

- Larger sample sizes to enhance the generalizability of the findings.
- Head-to-head comparisons to directly evaluate the relative efficacy of different therapeutic modalities, such as picosecond versus fractional ablative lasers or different standardized PRP preparations.
- The use of standardized and objective measures, including instrumental assessments like Cutometer^®^ for elasticity and quantitative histological analyses for neocollagenesis, to allow for reliable cross-study comparisons.
- Long-term follow-up to assess the durability of clinical and histological improvements over time.
- Greater demographic inclusivity by enrolling participants across a wider range of Fitzpatrick phototypes, particularly types V and VI, to validate efficacy and safety in currently underrepresented populations.

Implementing these rigorous methodological approaches will be fundamental to transforming the innovations discussed in this review into established, effective, and safe clinical protocols for the management of cutaneous photoaging.

## Figures and Tables

**Table 1:** Relevant information from the included studies (*n* = 22), including study design, population characteristics, treatment modalities, and main findings. Data is presented as reported in the original articles (Abbreviations: AAA, acetyl aspartic acid; AGE, trichloroacetic acid, pyruvic acid, salicylic acid, mandelic acid and lactobionic acid; AK, actinic keratosis; AMSC-CM, amniotic-derived mesenchymal stem cell conditioned medium; AMSC-MP, amniotic-derived mesenchymal stem cell metabolite product; COL-IV, collagen-IV; DLA, diffractive lens array; HA, hyaluronic acid; IFC-CAF, *Cryptomphalus aspersa* snail egg extract with skin stem cell activity; IGA, Investigator Global Assessment; MELA, mandelic acid, potassium azeloyl diglycinate, retinol, salicylic acid, phytic acid, lactobionic acid, and lactic acid; PDT, photodynamic therapy; PGA, Physician Global Assessment; PRGF, plasma-rich in growth factors; PRP, platelet-rich plasma; PSAL, picosecond pulsed alexandrite laser; RCM, reflectance confocal microscopy; RF, radiofrequency; SCA, *Cryptomphalus aspersa* secretion; SVF, stromal vascular fraction; and TEWL, transepidermal water loss).

Author, Year	Country	Study Design	Sample Size and Characteristics	Intervention Details	Key Findings
Bruce et al., 2016 [21]	USA	Non-randomized controlled trial	13 subjects, Fitzpatrick I–IV post chemical resurfacing	12-week continuation skin care post AGE/MELA peel	Maintenance/enhancement of resurfacing benefits; no adverse events.
Da Silva et al., 2021 [22]	Brazil	Non-randomized controlled trial with split-face design	19 women, mean age 54, Fitzpatrick I-IV, photoaging types II/III	Lyophilized PRP (2 months)	No significant improvement in skin aging with PRP
Díaz-Ley et al., 2015 [23]	Spain	Non-randomized, non-controlled (pre-post) experimental study	10 healthy volunteers (7F/3M), aged 34–59 years	Intradermal PRGF	Increased dermal/epidermal thickness; reduced solar elastosis; high satisfaction.
El-Domyati et al., 2025 [24]	Egypt	Non-randomized, non-controlled (pre-post) experimental study	12 subjects, Fitzpatrick III–IV, moderate photoaging	Microneedle + sublative fractional RF (3 sessions)	Biopsy-based analysis: increased collagen I and normalized elastic fibers; improved skin texture and wrinkles
Gillbro et al., 2015 [25]	Sweden	Non-randomized controlled trial	16 females (for each of two different trials), >55 years	Topical AAA 1% (12 days)	Increased COL-IV and fibrillin-1; improved skin firmness vs. vehicle; comparable to retinol.
Grippaudo et al., 2016 [26]	Italy	Randomized controlled trial	50 patients, Fitzpatrick II–V, photo/chronoaging	Fractional laser CO_2_ + ionic hydrogel (±antibiotics)	No difference between groups in healing time, post-operative complications and satisfaction; good adherence, no infections.
Indramaya et al., 2023 [27]	Indonesia	Non-randomized controlled trial	60 women	Microneedling vs. fractional CO_2_ laser + AMSC-CM + Vitamin C (3 sessions)	Fractional CO_2_ group superior on wrinkles, UV spots, pores
Jiménez et al., 2018 [28]	Spain	Randomized controlled trial with split-face design	10 female subjects, moderate-severe photoaging	YAG laser + regenerative serum vs. placebo	Superior epidermal/dermal regeneration and wrinkle reduction on the treated side; high satisfaction.
Lim et al., 2020 [29]	South Korea	Randomized controlled trial	50 women, 45–65 years, Fitzpatrick II–V, photoaging signs	SCA^®^ + IFC^®^-CAF vs. vehicle	Reduced TEWL, improved firmness, elasticity; superior PGA/IGA vs. placebo; similar wrinkle reduction in both groups
Liu et al., 2019 [30]	China	Randomized controlled trial with split-face design	22 participants, Fitzpatrick III-IV, photoaged facial skin	Microneedle fractional radiofrequency	Improved wrinkles, texture, and roughness score (Sa); minimal adverse events.
Makino et al., 2023 [31]	USA	Non-randomized, non-controlled (pre-post) experimental study	23 females, mean age 42.7, Fitzpatrick I–VI	Diamond-tip microdermabrasion + HA^5^-DG serum (5 different forms of HA)	Immediate and long-term improvements in hydration, fine lines, texture, and radiance
Marchlewicz et al., 2024 [32]	Poland	Randomized controlled trial	30 women, Fitzpatrick II-III	Microneedling or needle-free mesotherapy with 98.2% snail mucus	Histological improvements: increased collagen bundle thickness, dermal regeneration markers (Ki67, PCNA, MMP-2) improved
Mazur et al., 2023 [33]	Poland	Non-randomized, non-controlled (pre-post) experimental study	52 patients (34 women, 18 men) and 300 AK lesions, facial grade II AK, 275 non-pigmented, 25 pigmented	PDT	High remission rates of dermoscopic and RCM features, solar elastosis reduced
Prakoeswa et al., 2020 [34]	Indonesia	Randomized controlled trial	60 women, split group	AMSC-MP + Vitamin C vs. AMSC-MP alone, Dermapen^®^-assisted delivery (3 sessions)	Significant improvements in wrinkles, UV spots, pores in the combined group
Setyaningrum et al., 2021 [35]	Indonesia	Non-randomized, non-controlled (pre-post) experimental study	30 women, Glogau II–III	CO_2_ fractional laser + AMSC-CM + Vitamin C (3 sessions)	Significant wrinkle, pore, spot improvement; minor effect on skin tone
Signor et al., 2016 [36]	Brazil	Non-randomized controlled trial	10 patients (split group), Fitzpatrick I–V	SVF vs. calcium hydroxyapatite filler	Slight collagen increase in SVF group; no statistical differences; moderate satisfaction.
Truchuelo et al., 2020 [37]	Spain	Randomized controlled trial with split-face design	20 patients, split-face, moderate photoaging	Non-ablative fractional Laser (2 session) + SCA^®^ secretion 40% (daily for 28-days) vs. vehicle	SCA^®^ improved skin recovery post-laser, enhanced elasticity, reduced wrinkles and adverse effects
Widianingsih et al., 2019 [38]	Indonesia	Non-randomized controlled trial with split-face design	9 female participants, Glogau II–III	Fractional erbium laser + topical AMSC-MP	Improved pores, pigmentation; mixed wrinkle results; no serious adverse events
Wu et al., 2017 [39]	USA	Non-randomized, non-controlled (pre-post) experimental study	34 healthy subjects, 18–75 years, Fitzpatrick I–IV	Topical conjugated linolenic acid derived from pomegranate seed extract vs. dimethicone after fractionated ablative CO2-laser resurfacing	Faster resolution of itching, reduced edema; improved wrinkling and elastosis at day 14
Wu et al., 2016 [40]	USA	Non-randomized, non-controlled (pre-post) experimental study	20 subjects, Fitzpatrick I–IV	PSAL with DLA	Improved dyspigmentation, keratosis, texture; moderate satisfaction
Ye et al., 2023 [41]	China	Non-randomized, non-controlled (pre-post) experimental study	32 Chinese women, Fitzpatrick I–IV, mild photoaging	Supramolecular 0.1% retinol (twice-daily use)	Improved wrinkles, texture, elasticity, hydration, high tolerance
Yu et al., 2021 [42]	China	Randomized controlled trial with split-face design	10 women, 45–55 years, Fitzpatrick III–IV	PSAL with DLA	Sustained improvement in dyschromia, texture, and wrinkles up to 36 months, minimal adverse effects

## Data Availability

A template reporting the extracted data may be available upon motivated request and approval by the research team.

## Supplementary Materials

The following supporting information can be downloaded at https://www.mdpi.com/article/10.3390/biomedicines13112758/s1.

## Appendix A. Search Strategy

PubMed: (((“solar elastosis”[tiab] OR “cutaneous elastosis”[tiab] OR “photoaging elastosis”[tiab] OR “sun-induced elastosis”[tiab] OR elastosis[tiab]) AND (“damage prevention”[tiab] OR “photodamage prevention”[tiab] OR “injury prevention”[tiab] OR “preventive strategies”[tiab] OR prophylaxis[tiab])) AND (“regenerative medicine”[tiab] OR “stem cell therapy”[tiab] OR “cell therapy”[tiab] OR “new therapies”[tiab] OR “innovative therapies”[tiab] OR “tissue engineering”[tiab] OR regeneration[tiab]))) AND (“2014/01/01”[PDAT]:“3000/12/31”[PDAT]).

Scopus: ((TITLE-ABS-KEY(“solar elastosis”) OR TITLE-ABS-KEY(“cutaneous elastosis”) OR TITLE-ABS-KEY(“photoaging elastosis”) OR TITLE-ABS-KEY(“sun-induced elastosis”) OR TITLE-ABS-KEY(elastosis))AND(TITLE-ABS-KEY(“damage prevention”) OR TITLE-ABS-KEY(“photodamage prevention”) OR TITLE-ABS-KEY(“injury prevention”) OR TITLE-ABS-KEY(“preventive strategies”) OR TITLE-ABS-KEY(prophylaxis))AND(TITLE-ABS-KEY(“regenerative medicine”) OR TITLE-ABS-KEY(“stem cell therapy”) OR TITLE-ABS-KEY(“cell therapy”) OR TITLE-ABS-KEY(“new therapies”) OR TITLE-ABS-KEY(“innovative therapies”) OR TITLE-ABS-KEY(“tissue engineering”) OR TITLE-ABS-KEY(regeneration)))AND(PUBYEAR > 2013).

Web of Science: TS = (“solar elastosis” OR “cutaneous elastosis” OR “photoaging elastosis” OR “sun-induced elastosis” OR elastosis) AND TS = (“damage prevention” OR “photodamage prevention” OR “injury prevention” OR “preventive strategies” OR prophylaxis) AND TS = (“regenerative medicine” OR “stem cell therapy” OR “cell therapy” OR “new therapies” OR “innovative therapies” OR “tissue engineering” OR regeneration) AND PY = (2014–2025).

ProQuest: ((TI(“solar elastosis”) OR AB(“solar elastosis”) OR SU(“solar elastosis”) OR TI(“cutaneous elastosis”) OR AB(“cutaneous elastosis”) OR SU(“cutaneous elastosis”) OR TI(“photoaging elastosis”) OR AB(“photoaging elastosis”) OR SU(“photoaging elastosis”) OR TI(“sun-induced elastosis”) OR AB(“sun-induced elastosis”) OR SU(“sun-induced elastosis”) OR TI(elastosis) OR AB(elastosis) OR SU(elastosis)) AND (TI(“damage prevention”) OR AB(“damage prevention”) OR SU(“damage prevention”) OR TI(“photodamage prevention”) OR AB(“photodamage prevention”) OR SU(“photodamage prevention”) OR TI(“injury prevention”) OR AB(“injury prevention”) OR SU(“injury prevention”) OR TI(“preventive strategies”) OR AB(“preventive strategies”) OR SU(“preventive strategies”) OR TI(prophylaxis) OR AB(prophylaxis) OR SU(prophylaxis))) AND (TI(“regenerative medicine”) OR AB(“regenerative medicine”) OR SU(“regenerative medicine”) OR TI(“stem cell therapy”) OR AB(“stem cell therapy”) OR SU(“stem cell therapy”) OR TI(“cell therapy”) OR AB(“cell therapy”) OR SU(“cell therapy”) OR TI(“new therapies”) OR AB(“new therapies”) OR SU(“new therapies”) OR TI(“innovative therapies”) OR AB(“innovative therapies”) OR SU(“innovative therapies”) OR TI(“tissue engineering”) OR AB(“tissue engineering”) OR SU(“tissue engineering”) OR TI(regeneration) OR AB(regeneration) OR SU(regeneration))) AND PUBYEAR >= 2014.
