# Supplementary material for: Preventive and Therapeutic Interventions in Solar Elastosis and Photoaging: A Comprehensive Systematic Review
Source: Biomedicines. 2025 Nov 11;13(11):2758. doi: 10.3390/biomedicines13112758 (PMC12650578; doi:10.3390/biomedicines13112758)
Supplement: Supplementary file 1 [file biomedicines-13-02758-s001.zip › biomedicines-3912496-supplementary.pdf]

Table S1. Study selection process and records identified from each database

Database	Records identified	After year filter (≥2014)	After exclusion of reviews / irrelevant types	English language
**PubMed**	152	141	99	99
**Scopus**	404	380	211	196
**Web of Science**	162	152	116	—
**ProQuest**	59	57	—	—
**Total**	**777**	**730**	**426**	**295**

Table S2. Overall selection process

Screening phase	Number of records
Total records retrieved (all databases)	777
Duplicates removed	199
Records automatically excluded (irrelevant / ineligible)	342
Records screened	236
Records excluded after screening	202
Full-text articles assessed	34
Studies included in qualitative synthesis	22

Reasons for exclusion (n = 12)

- Wrong study design: 3
- Wrong outcome: 2
- Wrong population: 3
- Wrong intervention: 2
- Study protocol: 2
